# A real-time pluripotency reporter for the long-term and real-time monitoring of pluripotency changes in induced pluripotent stem cells

**DOI:** 10.18632/aging.204083

**Published:** 2022-05-15

**Authors:** Hong-Fen Shen, Yong-Long Li, Shi-Hao Huang, Jia-Wei Xia, Zhi-Fang Yao, Gao-Fang Xiao, Ying Zhou, Ying-Chun Li, Jun-Wen Shi, Xiao-Lin Lin, Wen-Tao Zhao, Yan Sun, Yu-Guang Tian, Jun-Shuang Jia, Dong Xiao

**Affiliations:** 1 Cancer Research Institute, School of Basic Medical Science, Southern Medical University, Guangzhou 510515, China; 2 Laboratory Animal Center, Southern Medical University, Guangzhou 510515, China; 3 Guangzhou Southern Medical Laboratory Animal Sci. and Tech. Co., Ltd., Guangzhou 510515, China; 4 The Third People’s Hospital of Kunming, The Sixth Affiliated Hospital of Dali University, Kunming 650041, China; 5 Akeso Biopharma, Inc., Zhongshan 528400, China; 6 Yue Bei People’s Hospital, Shaoguan 512025, China; 7 Department of Oncology, The First People’s Hospital of Chenzhou, Chenzhou 423000, China; 8 Cancer Center, Integrated Hospital of Traditional Chinese Medicine, Southern Medical University, Guangzhou 510315, China; 9 Department of Gastrointestinal Oncology, The Third Affiliated Hospital of Kunming Medical University, Yunnan Cancer Hospital, Yunnan Cancer Center, Kunming 650118, China; 10 Zhongshan School of Medicine, Sun Yat-Sen University, Guangzhou 510080, China; 11 National Demonstration Center for Experimental Education of Basic Medical Sciences, Southern Medical University, Guangzhou 510515, China

**Keywords:** induced pluripotent stem (iPS) cells, cancer cell reprogramming, cancer therapy, Oct4-EGFP transgene, pluripotency reporter system

## Abstract

To master the technology of reprogramming mouse somatic cells to induced pluripotent stem cells (iPSCs), which will lay a good foundation for setting up a technology platform on reprogramming human cancer cells into iPSCs. Mouse iPSCs (i.e., Oct4-GFP miPSCs) was successfully generated from mouse embryonic fibroblasts (MEFs) harboring Oct4-EGFP transgene by introducing four factors, Oct4, Sox2, c-Myc and Klf4, under mESC (Murine embryonic stem cells) culture conditions. Oct4-GFP miPSCs were similar to mESCs in morphology, proliferation, mESC-specific surface antigens and gene expression. Additionally, Oct4-GFP miPSCs could be cultured in suspension to form embryoid bodies (EBs) and differentiate into cell types of the three germ layers *in vitro*. Moreover, Oct4-GFP miPSCs could develop to teratoma and chimera *in vivo*. Unlike cell cycle distribution of MEFs, Oct4-GFP miPSCs are similar to mESCs in the cell cycle structure which consists of higher S phase and lower G1 phase. More importantly, our data demonstrated that MEFs harboring Oct4-EGFP transgene did not express GFP, until they were reprogrammed to the pluripotent stage (iPSCs), while the GFP expression was progressively lost when these pluripotent Oct4-GFP miPSCs exposed to EB-mediated differentiation conditions, suggesting the pluripotency of Oct4-GFP miPSCs can be real-time monitored over long periods of time via GFP assay. Altogether, our findings demonstrate that Oct4-GFP miPSC line is successfully established, which will lay a solid foundation for setting up a technology platform on reprogramming cancer cells into iPSCs. Furthermore, this pluripotency reporter system permits the long-term real-time monitoring of pluripotency changes in a live single-cell, and its progeny.

## INTRODUCTION

It is known that mouse and human cancer cells could be *in vitro* or *in vivo* reprogrammed into various cell types (including ESC-like cells and various differentiated cell types)(which were distinct from parental cells) by the *in vitro* and *in vivo* systems, such as 1) somatic nuclear transfer [1], 2) embryonic microenvironments (i.e., zebrafish embryos, chicken embryos and murine blastocysts) [2, 3], and 3) human embryonic stem cell (ESC) microenvironment [3], demonstrating that the gene expression profile and epigenetic state of these particular tumor cells were reversible upon their exposure to the above-mentioned microenvironments, but these *in vitro* and *in vivo* reprogramming systems are not applied to human clinical cancer therapy and the study of cancer cell reprogramming mechanisms because of technical constraints.

In recent years, induced pluripotent stem cells (iPSCs) have been *in vitro* generated from different cell types of several species, including mouse, rat, rabbit, sheep, pig, monkey and human, following ectopic expression of the transcription factors Oct4 and Sox2, combined with either Klf4 and c-Myc or Lin28 and Nanog [4–24], suggesting an *in vitro* powerful system and tool to *in vitro* examine the reprogramming mechanisms of somatic cells and cancer cells. The iPSC technology has also allowed iPSCs to be generated from tumor cells of various kind of cancers [i.e., gastrointestinal cancer [25], leukemia [26], melanoma [27, 28] and prostatic cancer [28], which provides us with the following invaluable information: 1) the epigenetic state of these particular tumor cells could be *in vitro* reversible by transcription-factor-mediated reprogramming; 2) the oncogenic mutations present in the reprogrammed tumor-derived iPSCs do not prevent the embryoid body (EB)-mediated differentiation and teratoma formation; 3) in contrast to natural cancer cells, these reprogrammed cells (including tumor-derived iPSCs and iPSC-derived differentiated cells) confer higher sensitivity to chemotherapeutic agents and differentiation-inducing treatments, suggesting the possibility of new cancer therapies via reprogramming approaches in cancer cells to induce susceptibility to therapeutic agents.

Nasopharyngeal carcinoma (NPC) is a high-incidence malignancy in Southern China and Southeast Asia, however, until now, the iPSC technology is not employed in the study of NPC cell reprogramming. Prior to assess whether transcription-factor-mediated reprogramming is equally suitable for reprogramming NPC cells into a pluripotent state, in this study we attempted to firstly convert mouse somatic cells into iPSCs, which will lay a solid foundation for setting up a technology platform on reprogramming cancer cells into iPSCs.

Furthermore, culturing and maintaining the pluripotent stem cells (i.e., ESCs and iPSCs) in an undifferentiated state is a tedious and expensive task, while spontaneous differentiation of ESCs and iPSCs is always observed in cell cultures [29–35]. Therefore, close monitoring the changing pluripotency of stem cells in live cells is essential for many studies [29–35]. The following assays for the pluripotency of stem cells, such as RT-PCR of ESC markers, immunofluorescence staining with stage-specific embryonic antigens, alkaline phosphatase activity and teratoma assay, can’t real-time monitor the pluripotency changes in live cells [8–17]. Here, we described a pluripotency monitoring system, in which the expression of enhanced green fluorescent protein (EGFP) is under the control of the promoter of a pluripotency gene (i.e., Oct4). This real-time pluripotency reporter system permits the long-term real-time monitoring pluripotency changes in a live single cell and its progeny.

## RESULTS

### Generation of Oct4-GFP miPSCs from mouse embryonic fibroblasts (MEFs)

**Figure 1:**
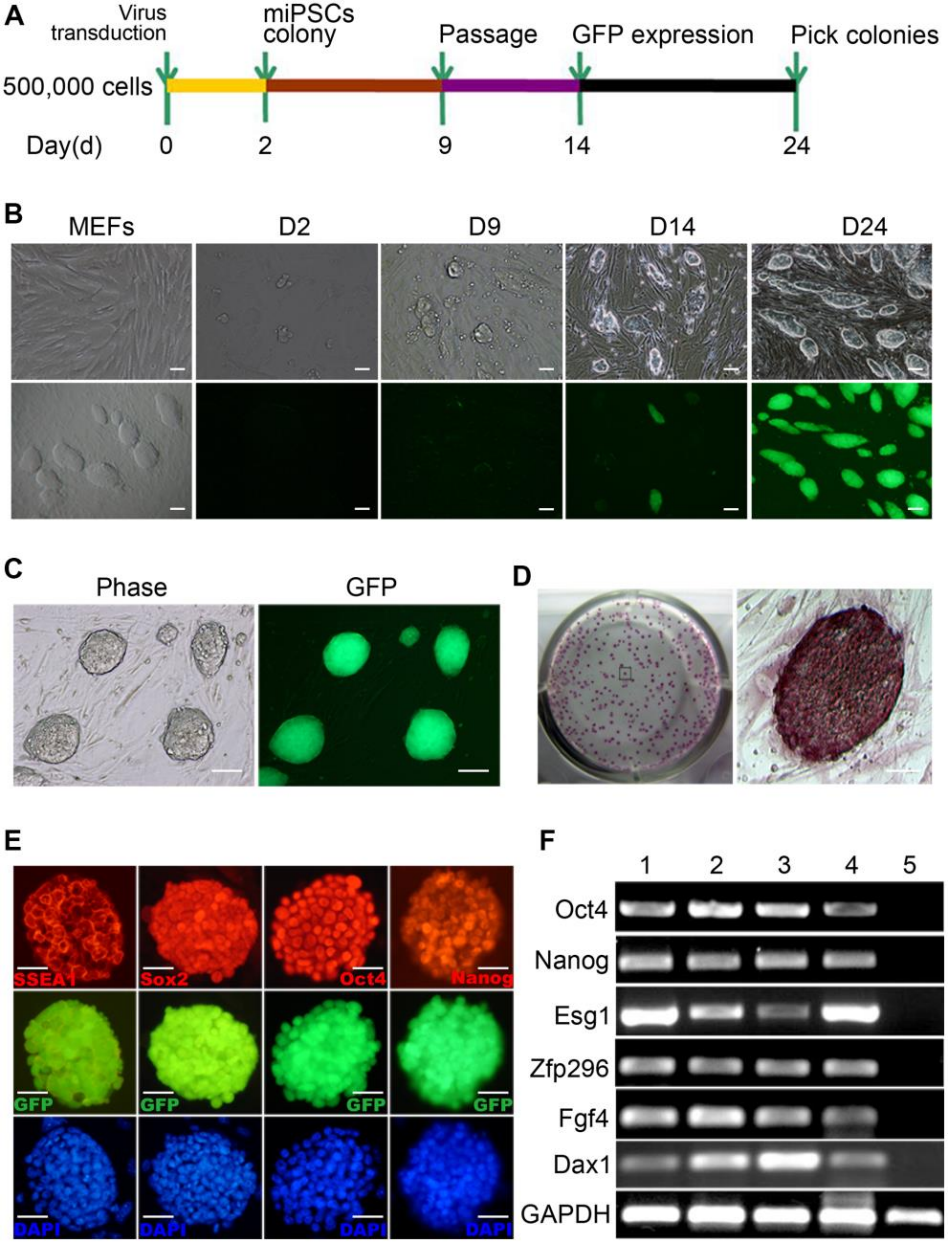
**Generation of mouse Oct4-EGFP iPSCs from mouse embryonic fibroblasts (MEFs).** (**A**) A schematic diagram of the reprogramming protocol used. (**B**) Typical Oct4-GFP^+^ miPSC colonies were initially observed around day 14. (**C**) The Oct4-GFP^+^ miPSCs sustain 30 generations and homogenous self-renewal under conventional mESC growth condition. (**D**) The long-term expanded iPSCs grow as compact and domed colonies that express strong alkaline phosphatase (ALP). (**E**) These miPSCs express typical pluripotency markers, and GFP (green) was shown to be colocalized with SSEA1(red), Sox2 (red), Oct4 (red) and Nanog(red). (**F**) RT-PCR analysis of endogenous pluripotency gene expression in Oct4-GFP^+^ miPSCs. Scale bar: (**B**, **C**) 100μm and (**D**, **E**) 50μm.

We introduced four genes (i.e., Oct4, Klf4, Sox2 and c-Myc) into MEFs from the homozygous Pou5f1-EGFP transgenic mouse embryos which contain an Oct4-GFP reporter by lentiviral transduction. Once the MEFs are reprogrammed, the GFP will be expressed and we can observe the reprogramming progress visually. Transduced cells were then cultured on irradiated ICR-MEFs feeder cells in ESC medium. 3 days after infection, some small clusters of cells without GFP expression emerged. At day 9, the cell mass grew up with GFP expression ambiguously at the edge. By day 14, a number of colonies with ESC-like morphology formed but only part of colonies has obvious GFP expression, then the colonies were picked and expanded into stable iPSC lines. After 24 day, all clones with typical ESC morphology express high GFP (Figure 1A, 1B). The Oct4-GFP^+^ miPSCs sustain long-term and homogenous self-renewal under conventional mESC growth condition (Figure 1C). The long-term expanded iPSCs grow as compact and domed colonies that express strong alkaline phosphatase (ALP) (Figure 1D). Furthermore, we chose the iPSC clones to examine whether typical pluripotency markers were expressed. As expected, further characterization of the primary iPSC clones revealed that they were positive for standard pluripotency markers such as SSEA1, Sox2, Oct4 and Nanog (Figure 1E), as determined by immunofluorescence. RT-PCR assay also demonstrated that they express pluripotency genes including Nanog, Oct4, Zfp296, Esg1, Dax1 and Fgf4 (Figure 1F). Collectively, these findings demonstrate that we have successfully reprogrammed MEFs to iPSCs.

### *In vitro* EB-mediated differentiation of Oct4-GFP miPSCs

**Figure 2:**
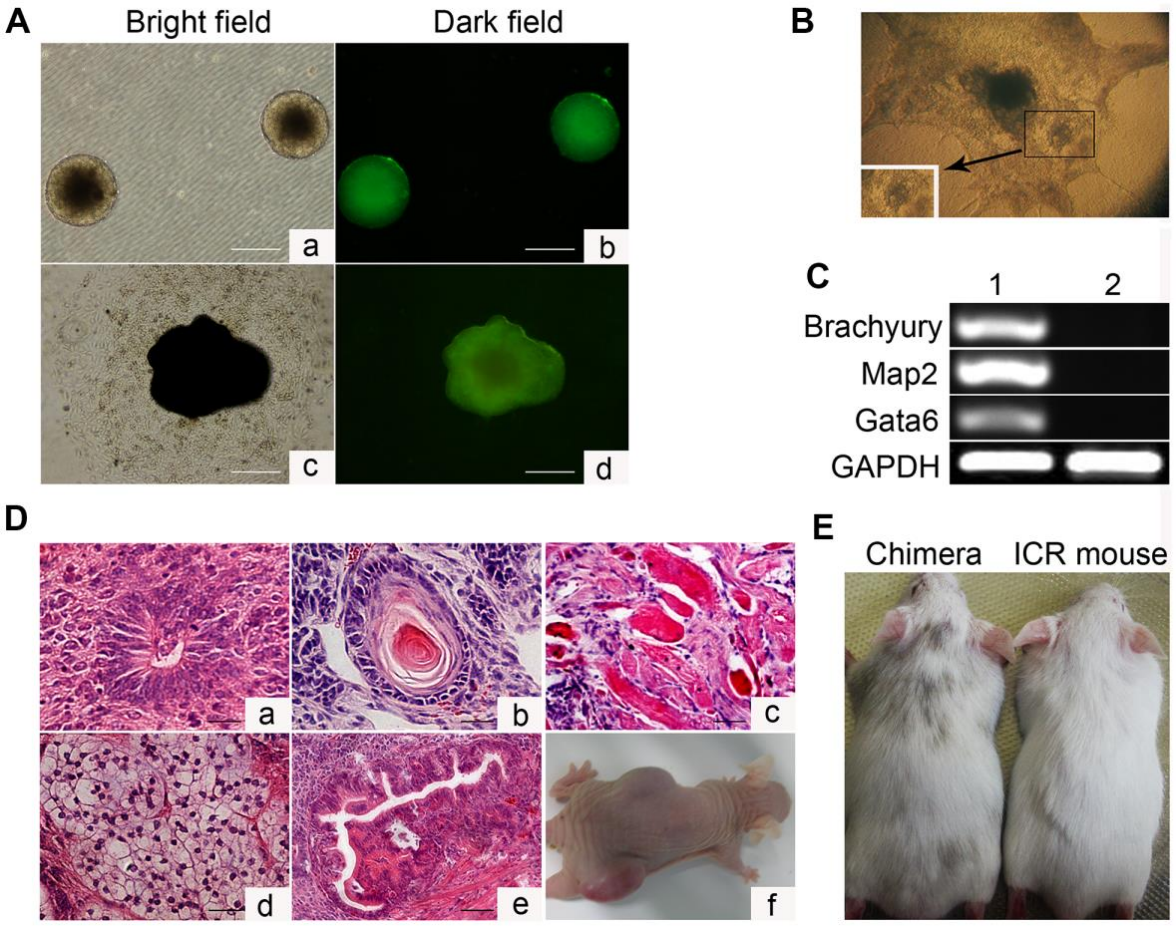
***In vitro* embryoid body-mediated differentiation and *in vivo* developmental pluripotency of Oct4-EGFP miPSCs.** (**A**) *In vitro* embryoid body (EB) formation (**a**, **b**) and differentiation (**c**, **d**). (**B**) *In vitro* EB differentiation into myocardium cells. (**C**) RT-PCR analyses of various differentiation markers for the following three germ layers in EB. Brachyury (a marker of mesoderm), microtubule associated protein 2 (Map2, ectoderm), and GATA-binding factor 6 (Gata6, endoderm). (**D**) Various tissues present in teratomas derived from Oct4-EGFP miPSCs. (**E**) Chimeric mouse generated by Oct4-EGFP miPSCs. Scale bar: (**A**, **D**) 50μm.

To characterize the *in vitro* differentiation ability of Oct4-GFP miPSCs, we first aggregated them into embryoid bodies (EBs) in suspension and then explanted them in adherent culture (Figure 2A). After 21 days, 3 out of 3 tested Oct4-GFP miPSC lines developed into contracting muscle fibers (“beating hearts”), suggesting the differentiation into the cardiomyocyte lineage (Figure 2B and Supplementary Movie 1). Additionally, total RNA was extracted from both Oct4-GFP miPSCs and harvested EBs, and then used to detect the expression of the indicated genes involved in development of each germ layer by RT-PCR. As expected, RT-PCR analysis showed that EBs expressed the genes of the ectoderm (Map2), endoderm (Gata6) and mesoderm (Brachyury) markers, whereas Oct4-GFP miPSCs did not express these genes (Figure 2C). Together, these results demonstrate that Oct4-GFP miPSCs display the multilineage differentiation potential *in vitro*.

### *In vivo* developmental pluripotency of Oct4-GFP miPSCs

Subsequently, we investigated the *in vivo* developmental potential of Oct4-GFP miPSCs by teratoma and chimera formation assays. Histological analysis of Oct4-GFP-iPSC-induced teratomas revealed that teratomas induced by these cells contained differentiated cell types representing all three embryonic germ layers, including neural tissues, cartilage, muscle tissues, adipose tissue and glandular epithelium (Figure 2D). Furthermore, Oct4-GFP miPSCs were injected into blastocysts to produce chimeric animals. The viable mice with coat color chimerism were efficiently generated, and could develop into adulthood (Figure 2E). Summarily, our results illustrate that Oct4-GFP miPSCs show pluripotent phenotype *in vivo*.

### The karyotype of Oct4-GFP miPSCs

**Figure 3:**
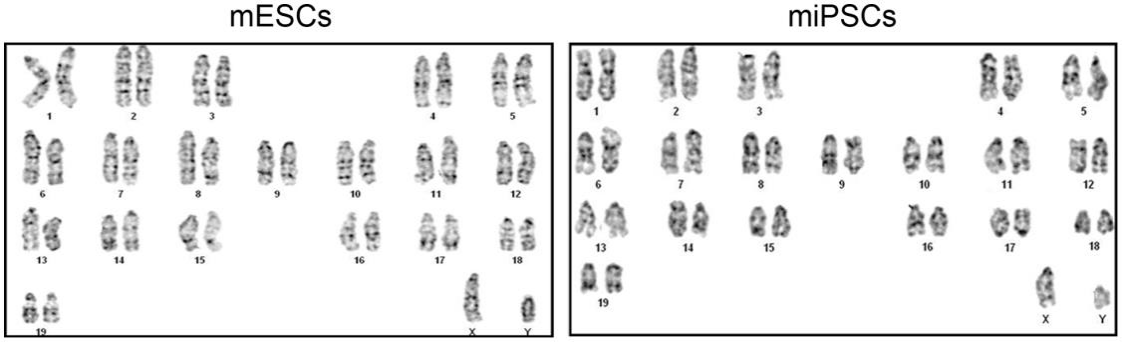
The normal karyotype of mESCs and Oct4-EGFP miPSCs.

The ES cells transmit genome through the germline of the chimeras, which depends on a normal chromosome number. Therefore, it is necessary to perform karyotyping analysis of Oct4-GFP miPSCs prior to the generation of chimeras. 1×10^6^ cells of every miPSC clone after 10 passages and ESCs were obtained separately at the time of splitting, and then karyotypic analyses were carried out according to published protocols. Our results revealed that compared to ESCs, some of Oct4-GFP miPSC clones had a normal 40 XY karyotype (Figure 3). Additionally, we found that some clones had an abnormal karyotypes comparing with ESCs, indicating that the proper chromosomal alterations of induced cells may be an ordinal event and probably interrupted by some error programming which needs to be further investigated.

### GFP expression associated with Oct4 and Sox2 expression during EB-mediated differentiation of Oct4-GFP miPSCs

**Figure 4:**
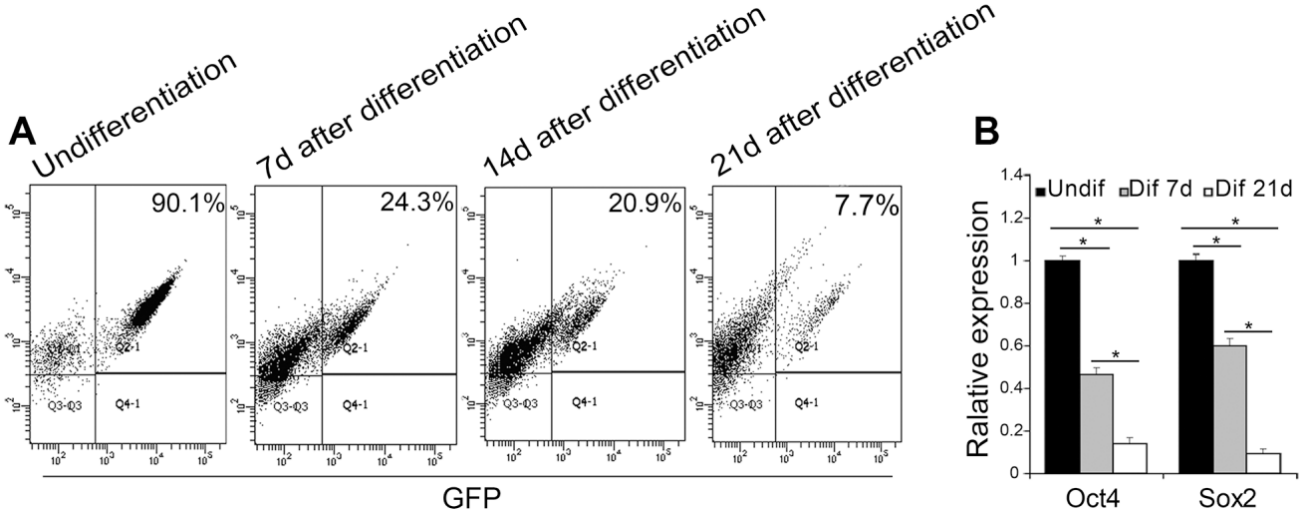
**EGFP expression associated with Oct4 and Sox2 expression during the EB-mediated differentiation of Oct4-EGFP miPSCs.** (**A**) The undifferentiated/differentiated cell state was assessed through GFP assay by flow cytometry (FCM) at 7th day, 14th day and 21st day after EB-mediated differentiation. (**B**) qRT-PCR analysis for the expression of ESC-specific transcription factors (i.e., Oct4 and Sox2) in Oct4-EGFP miPSCs and differential EBs.

In this study, EGFP reporter gene which is driven by the Oct4 promoter was employed to visually display the reprogramming process. To ascertain differentiation of Oct4-GFP miPSCs, we collected differential EBs at 7th day, 14th day and 21st day after culture, respectively, and to subsequently perform the analyses of FACS and qRT-PCR. FACS analysis revealed that when cultured *in vitro* continuously for 7, 14 or 21 weeks, EGFP positive rates of EBs were 24.3%, 20.9% and 7.7%, respectively, whereas EGFP-positive rate of Oct4-GFP miPSCs was approximately 90.1%, suggesting the EGFP-positive rate of EBs was more and more lower accompanying the differentiation of EBs (Figure 4A). Additionally, RT-PCR assay showed that the expression of Oct4 and Sox2 in differential EBs at 7th day and 21st day after culture dramatically decreased upon EB-mediated differentiation of Oct4-GFP miPSCs, compared with Oct4-GFP miPSCs (Figure 4B). Collectively, our findings demonstrate that GFP expression is coincident with the expression of Oct4 and Sox2 between Oct4-GFP miPSCs and EBs, indicating that we can preliminarily estimate the differentiation degrees of Oct4-GFP miPSCs by GFP assay under inverted fluorescence microscope.

### Cell cycle features of Oct4-GFP miPSCs

**Figure 5:**
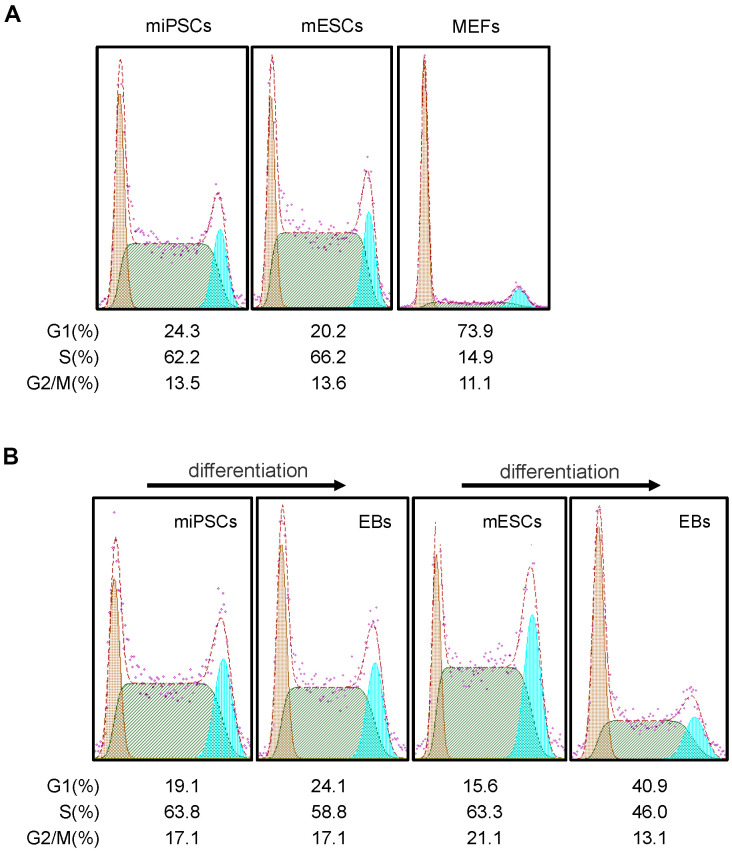
**Cell cycle features of Oct4-EGFP miPSCs.** (**A**) Comparison of cell cycle profiles between miPSCs, mESCs and MEFs. (**B**) miPSCs, mESCs and EBs derived from miPSCs or mESCs were stained with PI and analyzed by flow cytometry.

It is known that ESCs have an unusual cell cycle distribution which may relate to its stemness. To examine whether Oct4-GFP miPSCs have a similar feature of cell cycle distribution of ESCs, mouse ESCs (mESCs), Oct4-GFP miPSCs and MEFs were subjected to cell cycle analysis by FACS with propidium iodide (PI). FACS analysis of DNA content showed that the percentage of G1, S and G2/M phase cells was 20.2%, 66.2% and 13.6% (Figure 5A) or 15.6%, 63.3% and 21.1% (Figure 5B) in mESCs, and 24.3%, 62.2% and 13.5% (Figure 5A) or 19.1%, 63.8% and 17.1%(Figure 5B) in miPSCs, respectively, while the percentage of cells in G1, S and G2/M phases was 73.9%, 14.9% and 11.1% in MEFs, respectively. Together, our results reveal that unlike cell cycle distribution of MEFs, Oct4-GFP miPSCs are similar to mESCs in the cell cycle structure which consists of higher S phase and lower G1 phase.

Subsequently, we further detected the cell cycle distribution of EBs derived from differentiating mESCs and Oct4-GFP miPSCs, and we found that the percentage of G1, S and G2/M phase cells were 40.9%, 46% and 13.1% in mESC-derived EBs (Figure 5B), and 24.1%, 58.8% and 17.1% in miPSC-derived EBs (Figure 5B), respectively. After comparing cell cycle profiles between differentiated and undifferentiated cells, we observed that the trend of cell cycle distribution was similar for Oct4-GFP miPSCs and mESCs, whereas differentiated cells (i.e., EBs) was accompanied by obvious changes in the cell cycle distribution, characterized by an increase in the proportion of cells in G1 phase and a decrease in the proportion of cells in S phase.

## DISCUSSION

In this study, Oct4-GFP miPSCs were successfully generated from MEFs by introducing four factors, Oct4, Sox2, c-Myc and Klf4 under mESC culture conditions. Oct4-GFP miPSCs were similar to mESCs in morphology, proliferation, mESC-specific surface antigens and gene expression. Additionally, Oct4-GFP miPSCs could be cultured in suspension to form EBs and differentiate into cell types of the three germ layers *in vitro*. Moreover, Oct4-GFP miPSCs could develop to teratoma and chimera *in vivo*. Unlike cell cycle distribution of MEFs, Oct4-GFP miPSCs are similar to mESCs in the cell cycle structure which consists of higher S phase and lower G1 phase. Summarily, Oct4-EGFP miPSC line was successfully produced from MEFs.

These pluripotent stem cells not only have great potential for regeneration medicine, but also provide the opportunity to study and understand human development as they have the capacity to differentiate into all three germ layer-derived cells and are syngeneic they have an unlimited capacity for self-renewal and can differentiate into derivatives of all three embryonic germ layers (i.e., ectoderm, mesoderm and endoderm) [8–17]. Developing a better understanding of the underlying mechanisms of pluripotency and differentiation is pretty important, because it can provide knowledge that will allow specific manipulation of the development of human cells, to direct stem cells for generating specific cells types, and to understand abnormal cell development in conditions such as cancers [8–17].

Various experimental techniques, including somatic cell nuclear transfer [1], human embryonic stem cell (ESC) microenvironment [3], cell fusion [36] and somatic cell reprogramming with defined transcription factors [6, 7], have been developed for deriving pluripotent stem cells. Many routing protocols have been developed to maintain the self-renewal of stem cells [8–17]. However, maintaining the pluripotent stem cells in culture is a tedious and expensive task. Spontaneous differentiation is always observed in stem cell cultures, and pluripotent stem cells are maintained by removing such differentiated cells during serial passage. Therefore, close monitoring the changing pluripotency of stem cells in culture is required for many studies [29–35].

Pluripotency of stem cells can be identified by RT-PCR of ESC markers (i.e. Oct4, Sox2 and Nanog, immunofluorescence staining with stage-specific embryonic antigens (Oct4, Sox2, Nanog, SSEA-3, SSEA-4, TRA-l-60 and TRA-1-81), alkaline phosphatase activity and teratoma assay [8–17]. However, these approaches often involve destruction or fixation of cells during assay, and can’t be employed to real-time monitor the pluripotency of live cells [29–35]. To real-time monitor the pluripotency of live cells, various promoters have been trialled for their ability to drive expression specifically in pluripotent cells [29–35], and the artificial Oct4 promoter sequences-based promoter driving expression of EGFP appears to provide the highest efficiency and specificity in human ESCs and iPSCs [29, 30]. Zhong et al. had developed a pluripotency monitoring system in which the expression of EGFP is under the control of the promoter of a pluripotency gene (Rex-1) [31]. Wiraja et al. reported the utilization of nanosensor platform to allow facile, nonintegrative monitoring of cellular reprogramming processes *in situ* within live cells [35]. Here we have described the successful development of a real-time pluripotency reporter for the monitoring any changes in the pluripotency of Oct4-GFP miPSCs in real time and at single-cell resolution. Additionally, human ESCs and iPSCs have been stably modified with an artificial EOS(C3+) promoter driving expression of EGFP and puromycin resistance-conferring proteins, while EGFP expression faithfully reports on the pluripotency status of the cells in these lines, and that antibiotic selection allows for an efficient elimination of differentiated cells from the cultures [34]. Collectively, the optimization of pluripotent stem cell expansion and differentiation is facilitated by biological tools that permit non-invasive and dynamic monitoring of pluripotency. More importantly, use of these mentioned-above reporter systems will facilitate the study of stem cell pluripotency at the single-cell level, and sheds light on the molecular mechanisms of stem cell self-renewal and subsequent differentiation.

In conclusion, our findings demonstrate that Oct4-GFP miPSC line is successfully generated, which will lay a solid foundation for setting up a technology platform on reprogramming cancer cells into iPSCs. More importantly, this pluripotency reporter system facilitates the long-term real-time monitoring of pluripotency changes in a live single-cell, and its progeny.

## MATERIALS AND METHODS

### Mice

The homozygous Pou5f1-EGFP transgenic mice (B6;CBA-Tg(Pou5f1-EGFP)2Mnn/J; Stock Number: 004654) [37] were obtained from Model Animal Research Center of Nanjing University. The wild-type ICR mice were obtained from Cyagen Biosciences (Guangzhou, China) Inc. Nude mice were purchased from Center of Experimental Animals, Southern Medical University. All animal care and experimentation were performed according to the Study and Ethical Guidelines for Animal Care, handling and termination established by the Subcommittee of Southern Medical University on laboratory animal care. The presented work was approved by the ethical committee of Southern Medical University and is covered by Chinese animal husbandry legislation.

### Primary culture of Oct4-EGFP reporter MEFs used for iPSC induction

MEFs harboring Oct4-EGFP transgene (Oct4-EGFP-MEFs) were isolated from 13.5 d.p.c. embryos from the homozygous Pou5f1-EGFP transgenic mice.

### Lentiviral production and concentration

The single lentiviral vector of pHAGE2-EF1α-STEMMCA expressing a “stem cell cassette” composed of the four transcription factors (i.e., Oct4, Klf4, Sox2 and c-Myc) [38] was generously provided by Dr. Gustavo Mostoslavsky. Lentiviral production was performed as described previously [39, 40]. Briefly, lentiviruses were prepared by co-transfecting packaging vectors (psPAX2 and pMD2.G obtained from Torono Lab) with pHAGE2-EF1α-STEMMCA into 293FT cells (ATCC) using Lipofectamine 2000 (Invitrogen). Viral supernatants were collected at 72 hours (h), and subsequently filtered through 0.45 μm pore size cellulose acetate filters (Millipore), followed by ultracentrifugation at 50000 g at 4° C for 90 min for concentration. Viral stocks were stored at −80° C until transduction.

### Lentivirus transduction and iPSC induction

Oct4-EGFP reporter MEFs harboring an EGFP reporter gene were transduced by the concentrated viruses (carrying the above-mentioned stem cell cassette) supplemented with 5 mg/ml polybrene (Sigma) for 12h, and then incubated with mESC culture medium supplemented with LIF(Millipore) for 2.5 days. 3 days after infection, the dissociated Oct4-EGFP reporter MEFs were replated onto irradiated ICR-MEFs and cultured in mESC culture medium supplemented with LIF for the following days. The fresh medium was changed every other day. 14 days after transduction, some colonies with GFP expression emerged. The colony morphologies were similar to that of mouse ES cells. We extracted colonies into an MEF feeder and cultured the iPSCs according to the standard mouse ES cell culture protocol.

### Alkaline phosphatase staining and immunofluorescence staining

Alkaline phosphatase (AP) staining was performed according to the manufacturer’s protocol by using the Alkaline Phosphatase Detection Kit (#SCR004, Millipore). The iPSCs were cultured for five days at low to medium density, and then were fixed in 4% paraformaldehyde for 1-2 min. After aspirating the fixative, iPSCs were rinsed with TBST, and subsequently staining solution was added to cover each well in dark at room temperature (RT) for 15 min. The staining solution was subsequently discarded, and the cells were rinsed with TBST and then covered with 1x phosphate-buffered saline (PBS) to prevent drying. Finally, the number of AP-positive colonies was counted.

For immunofluorescence assay, cells were fixed in 4% paraformaldehyde for 30 min at RT, after washing with PBS, the fixed cells were then blocked for 30 min at RT with PBS containing 10% bovine serum albumin (BSA; Sigma-Aldrich) and 0.1% Triton X-100. The cells were then incubated with primary antibody overnight at 4° C in blocking buffer. Next day, cells were washed with PBS and incubated with fluorescently coupled secondary antibody in PBS containing 0.1% Triton X-100 for 1h at RT. The nuclei were stained with DAPI (Sigma) for 3 min at RT. All images were captured using an inverted fluorescence microscope (Nikon Eclipse TE2000-U). Antibody information is provided in Supplementary Table 1.

### Hematoxylin-eosin staining

For hematoxylin-eosin (HE) staining of tissues, 5μm paraffin-embedded sections were prepared from teratoma and incubated in citrate buffer (pH 6.0) at 92° C for 20 min. The sections were then washed three times with PBS. HE staining of teratomas sections was performed according to the manufacturer’s guidelines.

### RNA extraction, RT-PCR and qRT-PCR

Total RNA was extracted by using Trizol reagent (TaKaRa) and treated with DNase I to remove genomic DNA contamination, and then transcribed into cDNA by using oligo(dT) primer and the PrimeScript RT reagent Kit (TaKaRa), according to the manufacturer’s instructions. For reverse transcription PCR (RT-PCR), samples were performed with gene-specific primers and Taq polymerase (TaKaRa) and amplified in a thermocycler. For quantitative real-time PCR (qRT-PCR), each sample was performed using SYBR Green qRT-PCR master mix (TaKaRa) and analyzed in triplicate with GAPDH as the inner control. Amplification data were collected using the Stratagene Mx3005P. The list of primers is provided in Supplementary Tables 2–4.

### Karyotyping analysis

Karyotypes of Oct4-EGFP iPSCs and mESCs were determined by the standard method at Center for Prenatal and Hereditary Disease Diagnosis, Nanfang Hospital, Guangzhou 510515, China. Oct4-EGFP iPSCs and mESCs were treated with colchicine, then dissociated by trypsinization and centrifuged to be collected. Hypotonic shock was performed with 75mM KCI for 25 min at 37° C. The fixative used was methanol: glacial acetic acid (3: 1, v: v) with 2 changes at 20-min intervals. The preparation remained in the last change for at least 12 h. Slides were conventionally prepared and stained with Giemsa solution.

### *In vitro* EB-mediated differentiation of Oct4-EGFP iPSCs

*In vitro* the differentiation of Oct4-EGFP iPSCs was carried out by the standard EB differentiation method. The iPSCs were dissociated by trypsinization, and then cultured in ultra-low attachment 100-mm dish in the ES medium without LIF to form EBs. The medium was changed every other day. After 7 days of suspension in culture, the aggregated EBs were harvested and transferred to gelatin-coated plate and cultured in the same medium for another 7 days. Total RNA derived from plated EBs was used for RT-PCR analysis.

### Teratoma formation and histological analysis

iPSCs (1×10^6^ cells) were injected subcutaneously into each dorsal flank of recipient nude mice. Four weeks after the injection, teratoma were surgically dissected from mice. Teratoma specimens were fixed in PBS containing 4% formaldehyde, and embedded in paraffin. Paraffin sections were stained with HE. All animal experiments were performed in accordance with institutional guidelines.

### Chimera generation

Prior to iPSC injection, 3.5 day blastocysts derived from ICR mice were incubated in KSOM medium (EmbryoMax, Chemicon) at 37° C and 5% CO_2_. Blastocysts were injected with 10 to 15 Oct4-EGFP iPSCs and transferred into the uterine horn of pseudopregnant (2.5 dpc) 6- to 8-week-old ICR mice.

## Supplementary Material

Supplementary Tables

Supplementary Movie 1
