# Supplementary material for: A real-time pluripotency reporter for the long-term and real-time monitoring of pluripotency changes in induced pluripotent stem cells
Source: Aging (Albany NY). 2022 May 15;14(10):4445–58. doi: 10.18632/aging.204083 (PMC9186763; doi:10.18632/aging.204083)
Supplement: Supplementary Tables [file aging-14-204083-s001.pdf]

SUPPLEMENTARY TABLES

Supplementary Table 1. List of antibodies and suppliers used for immunofluorescence.

Antibody	Isotype	Suppliers
Oct4	Rabbit IgG1	Abcam
Sox2	Rabbit IgG1	Abcam
Nanog	Rabbit IgG1	Abcam
SSEA1	Rabbit IgG1	Abcam

Supplementary Table 2. Primers for RT-PCR analysis of iPS cells.

Gene	Forward primer (5'-3')	Reverse primer (5'-3')	Accession
Oct4	CTGAGGGCCAGGCAGGAGCACGAG	CTGTAGGGAGGGCTTCGGGCACTT	NM_013633
Nanog	AGGGTCTGCTACTGAGATGCTCTG	CAACCACTGGTTTTTCTGCCACCG	NM_028016
Esg1	GAAGTCTGGTTCCTTGGCAGGATG	ACTCGATACACTGGCCTAGC	NM_025274
Fgf4	CGTGGTGAGCATCTTCGGAGTGG	CCTTCTTGGTCCGCCCGTTCTTA	NM_010202
Dax1	TGCTGCGGTCCAGGCCATCAAGAG	GGGCACTGTTCAGTTCAGCGGATC	NM_007430
GAPDH	ATCCCAGAGCTGAACGGGAA	TGCTTCACCACCTTCTTGATG	NM_008084
Zfp296	CCATTAGGGGCCATCATCGCTTTC	CACTGCTCACTGGAGGGGGCTTGC	BC099454

Supplementary Table 3. Primers for RT-PCR analysis of differentiated EBs.

Gene	Forward primer (5'-3')	Reverse primer (5'-3')	Accession
Brachyury	ATGCCAAAGAAAGAAACGAC	AGAGGCTGTAGAACATGATT	NM_009309
Map2	CCTCAGAACAAACAGCCACA	ATGCCAGATTTGGGGGTT	NM_001039934.1
GAPDH	ATCCCAGAGCTGAACGGGAA	TGCTTCACCACCTTCTTGATG	NM_008084
Gata6	ACCTTATGGCGTAGAAATGCTGAGGGTG	CTGAATACTTGAGGTCACTGTTCTCGG	NM_010258.3

Supplementary Table 4. Primers for qRT-PCR analysis of differentiated EBs.

Gene	Forward primer (5'-3')	Reverse primer (5'-3')	Accession
Oct4	TGCTGAAGCAGAAGAGGATCA	TGTTCTTAAGGCTGAGCTGCA	NM_013633
Sox2	TGAACGCCTTCATGGTATGGT	TTCTCGGTCTCGGACAAAAGT	NM_011443
GAPDH	ATCCCAGAGCTGAACGGGAA	TGCTTCACCACCTTCTTGATG	NM_008084
